# Insights into nanoparticle shape transformation by energetic ions

**DOI:** 10.1038/s41598-023-33152-9

**Published:** 2023-04-18

**Authors:** Aleksi A. Leino, Ville E. Jantunen, Pablo Mota-Santiago, Patrick Kluth, Flyura Djurabekova

**Affiliations:** 1 Helsinki Institute of Physics and Department of Physics, University of Helsinki, P.O. Box 43, FI-00014 Helsinki, Finland; 2 MAX IV Laboratory, Lund University, P.O. Box 118, SE-22100 Lund, Sweden; 3 Department of Materials Physics, Research School of Physics, Australian National University, Canberra, ACT 2601 Australia

**Keywords:** Nanoscience and technology, Nanoscale materials, Nanoparticles, Synthesis and processing, Materials science, Computational methods

## Abstract

Shape modification of embedded nanoparticles can be achieved by means of swift heavy ion irradiation. During irradiation, the particles elongate and align with the direction of the ion beam, presumably due to nanometer-scale phase transitions induced by individual ion impacts. However, the details of this transformation are not fully understood. The shape of metal nanoparticles embedded in dielectric matrices defines the non-linear optical properties of the composite material. Therefore, understanding the transformation process better is beneficial for producing materials with the desired optical properties. We study the elongation mechanism of gold nanoparticles using atomistic simulations. Here we focus on long-timescale processes and adhesion between the nanoparticle and the matrix. Without the necessity of *ad-hoc* assumptions used earlier, our simulations show that, due to adhesion with the oxide, the nanoparticles can grow in aspect ratio while in the molten state even after silicon dioxide solidifies. Moreover, they demonstrate the active role of the matrix: Only explicit simulations of ion impacts around the embedded nanoparticle provide the mechanism for continuous elongation up to experimental values of aspect ratio. Experimental transmission electron microscopy micrographs of nanoparticles after high-fluence irradiation support the simulations. The elongated nanoparticles in experiments and their interface structures with silica, as characterized by the micrographs, are consistent with the simulations. These findings bring ion beam technology forward as a precise tool for shaping embedded nanostructures for various optical applications.

## Introduction

Ion irradiation is a versatile tool for analyzing and modifying materials. When ions are accelerated to kinetic energies exceeding about 1 MeV/u, they reach the regime of swift heavy ions (SHIs). In this regime, the interactions of accelerated ions with the target electrons become the primary cause of their deceleration in the material. The effects of SHIs differ from that of ions in the keV -regime. In particular, most effects induced by individual ions are confined within cylindrical regions of several microns in length but only a few nanometers in diameter^1^.

This feature of SHIs can be used for modification of fine nanoscale structures. For instance, it was shown that SHI irradiation can cause a peculiar shape transformation of nanoparticles (NPs) embedded in amorphous silica matrices. Numerous experiments^2–13^ have shown that spherical NPs can become elongated in the direction of the ion beam and eventually evolve into prolate shapes or rods. While intriguing from an application point of view, the shaping process also offers unique insights into the fundamentals of ion-solid interactions. Consequently, the phenomenon has been extensively researched^14^ since the first reports^2^. From an application point of view, the process produces large arrays of aligned NPs embedded within a solid. It has technological relevance since, for example, the shape of the NPs – controlled by the irradiation parameters – affects the collective response of the composite material to external electromagnetic radiation. This control is essential in plasmonics research, whose potential applications range from sensing and imaging devices^15^ to building subwavelength photonic circuits^16^. However, no complete theoretical description of the shaping process has been developed so far, and even the primary mechanism by which the transformation occurs is still subject to debate.

Building a theoretical description has been particularly challenging as many details related to the SHI collision kinetics and the relaxation pathways of the initial, highly excited electronic subsystem are still not well understood. Quantitative analysis usually invokes the two-temperature model (inelastic thermal spike model), Coulomb explosion model, and the exciton self-trapping model to explain the experimental observations^17^. Due to the empirical parameters present in the models, it has not been possible to distinguish between them. While most first-principles approaches^18,19^ are still limited to studying femtosecond dynamics, Monte Carlo^20,21^ simulations describing the initial kinetics of the electrons and holes can reach longer time and spatial scales. Recently, these have been combined with molecular dynamics (MD) simulations^22^. Such approaches are promising, but the number of cases studied extensively is still low. Moreover, describing relevant phases accurately with the interatomic potentials and comparing the modeling results to experiments poses a formidable challenge. Regardless, SHI effects can often be successfully explained by rapid heating of the lattice in cylindrical symmetry irrespective of the underlying theoretical model invoked to explain the heating^23,24^. Consequently, simulating rapid, cylindrical heating in classical MD simulations (which can be implemented simply by increasing the atom velocities near the ion trajectory) provides a powerful tool to understand the later stages of a SHI impact^25,26^ even without an accurate description for the initial radial distribution of deposited kinetic energy.

Accordingly, the elongation phenomenon has been mostly explained in terms of heating effects. Two prominent models have emerged along this vein. The first considers the ion-hammering effect, which, due to the combined effect of many transient, molten ion tracks, causes strain in the matrix perpendicularly to the beam^23,24^. This effect occurs above a threshold electronic stopping power and fluence in amorphisable materials and generates perpendicular stresses within the NPs^24^. These stresses have been speculated to shape the nanoparticle^3,10,27^ when combined with the irradiation-induced softening or melting of the NP. The second class of explanations focuses on the high pressure generated within the NP by individual SHI impacts^2,9,24,28^. A small elongation increment occurs after each impact on the NP. In a scenario supported by MD simulations^9,29^, the molten and expanding material upon the ion impact pushes from the NPs to the underdense, molten track on top and beneath the nanoparticle. Silica remains solid elsewhere when the ion trajectory intersects the NP at the center region. On the other hand, it has been calculated that impacts at the sides of a sufficiently large NP do not heat the latter above the melting point^10,30^. This explains the alignment of the elongation process with the beam direction. Additionally, the ejection of atoms from the NP due to SHIs^2,3,28^ and spin-spin interactions^31^ have been considered to contribute to the shape change. The ion hammering scenario gains support from the observation of no shape modification in metal NPs embedded in small (shell thickness smaller than 26 nm) colloidal silica particles^3^. NPs embedded in NPs with a thicker shell, however, were found to elongate^3^. A contrary evidence reported a small shape modification for metal NPs irradiated at a fluence below the threshold values for ion hammering phenomenon. This evidence indicates that deformation is caused by individual ion impacts^32^. Moreover, theoretical continuum mechanical considerations do not support the idea of deformation by ion hammering, either^24^. Recent works include the observation of elongation using C${}_{60}$ ions^12^, studies on embedded NPs at the interface of silica and silicon nitride^11^, and the observation of elongation in NPs embedded in crystalline indium tin oxide^13^. See for example, Refs.^14,33,34^ for more complete reviews on the elongation phenomenon.

Atomistic simulations performed by several groups have shown that NPs change shape on impacts^9,28,29,35^. On the other hand, the mere reproduction of the effect in the simulation does not unambiguously identify the underlying mechanism, and the conclusions drawn from MD simulations appear contradictory. The authors in Ref.^28^ attribute the elongation to the formation of a shock wave, extremely high temperatures of the NP (9000 K), and associated interfacial pressures. On the other hand, other authors^9,29,35^ contribute the driving force to be the volume expansion due to melting and heating that occurs in considerably lower temperatures (2000 K). Using first-principles MD computations, authors in Ref.^31^ suggested that spin-effects play a central role in the elongation. Additionally, the effect of interparticle diffusion has been investigated using the lattice kinetic Monte Carlo method^36^.

Depending on the NP size and the ion modeled, the widely used two-temperature model typically predicts initial NP temperatures in the range of 1500–3000 K^5,6,30,37^. However, the accuracy of this model is still a topic of ongoing research and discussion^38^. The occurrence of the elongation with broad range of initial NP temperatures used in MD simulations^9,28,39^ indicates the robustness of the effect and certain insensitivity to the theoretical models used to implement the effect of SHI. While the details of the specific model used in the simulations may be important for quantitative predictions, they are not critical for understanding the driving force of the elongation phenomenon qualitatively. For example, the radial energy deposition profile (how much the velocities are increased as a function of distance to the ion trajectory) to silicon dioxide can be truncated, broadened, weakened, or changed in shape completely^9,28,39^ while still obtaining elongation. However, not all energy depositions result in elongation. Central requirements for elongation are: (1) sufficiently high total energy deposition so that at least a transient underdense ion track forms in the host matrix^40^ (2) the resulting ion track is smaller than or comparable to the size of the NP^9^ (3) the NP melts at least partially^39^. The duration of the molten, underdense ion track in the host matrix (some tens of ps) defines a time window for the molten NP material to expand, while the temperature of the NP sets the speed of this expansion (the elongation rate)^39^. Unfortunately, a fully quantitative model is not yet feasible as many parameters regarding the processes at the interface may affect the temperature of the NP (and hence the elongation rate) are not yet known^37^. Moreover, ion flux or interparticle diffusion might also be important, but cannot be captured within the short timescales of MD simulations. With the typical fluxes used experimentally (e.g. 10${}^{10}$–10${}^{11}$ ions/cm${}^2$ s in Ref.^6^), the time between impacts to the particle is in the order of seconds, while typical MD simulations of an ion impact are in the order of sub-ns^14,28,39^. In addition, some interatomic potentials can overestimate the melting point of the matrix material, which affects the simulated ion track sizes^9^.

In this article, we study the role of the silica matrix using classical MD simulations in the ion shaping of Au NPs in order to understand the elongation mechanism and bridge the gap to quantitative models. We introduce a description of Au-silica interactions using density functional theory (DFT) and show that the aspect ratio of the NP can change even after the ion track has solidified due to the adhesive forces between gold and silica. Furthermore, the evolution of the NPs between subsequent impacts was either neglected or heavily simplified in previous works. Here we demonstrate that secondary processes, i. e. indirect impacts and relaxation of the NP, play a significant role in the shape transformation.

We present the results of long-scale MD simulations that reveal an amorphous-to-crystalline phase transition and the build-up of negative stress in the NP within the first six ns after the impact. We show that this stress is relaxed by indirect impacts, which allows the elongation process to continue. The results suggest that both the NP and the matrix play an active role in the transformation. Moreover, we present high-resolution transmission electron microscope (HR-TEM) micrographs of NPs elongated due to high-fluence irradiation that confirm the polycrystalline structure of the NPs and show similar metal-matrix interface characteristics as predicted by the simulations.

## Results and discussion

### Interatomic potential for the Au-silica system

Previous studies on the amorphous silica-Au system using first-principles simulation methods have revealed a high diffusion barrier for gold through silica rings^41^, weak binding^41–43^ to regular sites, surface dewetting^43^, and that the interface does not intermix^43^. Hence in the previous simulations^9,39^ of Au NP elongation, we used a universal, purely repulsive pair potential to describe interatomic interactions at the interface as a first approximation.

**Figure 1:**
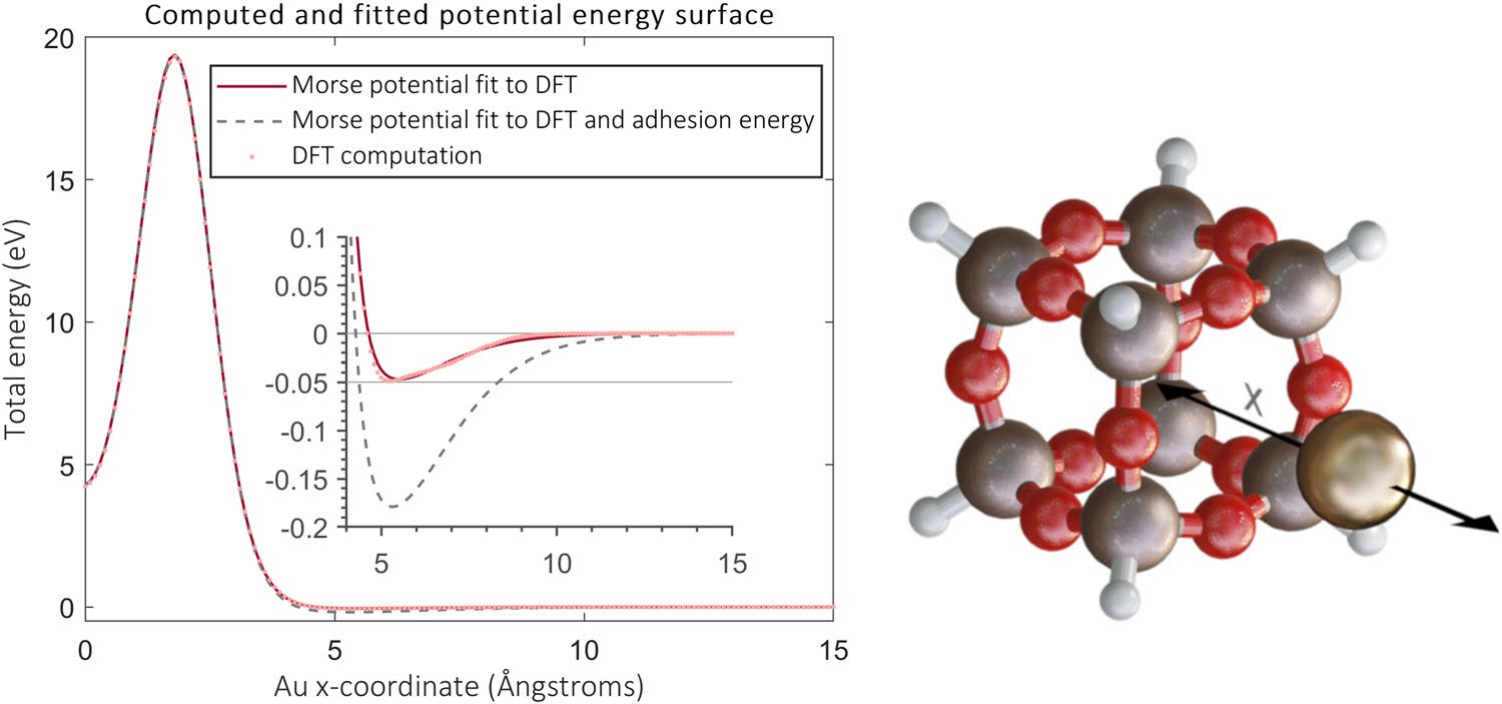
Potential energy surface scan for the Au-H${}_{\text {8}}$Si${}_8$O${}_{12}$ system. The graph on the left shows the data points obtained from the scan, and the Morse potential fits. The inset shows a magnification. The solid line indicates the original potential and the dashed shows the potential after fitting to adhesive energy as explained in the “Methods” Section. The figure on the right shows the geometry of the relaxed molecule and illustrates the x-coordinate. x = 0 points to the middle of the molecule.

As pointed out by Klaümunzer^24^, the interfacial energies (energy cost to form the surface) in metal-ceramics are in the order of 1 J/m${}^2$, which can cause interfacial pressures in metal NPs comparable to those from the ion hammering effect. The adhesive energy (the cost to separate the surfaces) of the Au-silica surface is several hundreds of mJ/m${}^2$ in magnitude^43,44^ but not previously taken into account. Hence, we introduce a Morse-type potential that describes the interface adhesion and the diffusion barrier through silica rings by fitting it to the energies predicted by a DFT computation and the experimental value of adhesive energy. Following Ref.^42^, we base our pair potential on a octasilasesquioxane (H${}_{\text {8}}$Si${}_8$O${}_{12}$) -molecule, shown in Fig. 1, to reduce the computational cost.

We use hydrogen atoms to terminate the bonds of Si atoms so that the structure represents a defect-free region of silica. We obtain the Morse potential by fitting it to the potential energy curve obtained by dragging a gold atom through a silica ring, as indicated in Fig. 1. The curve shows that gold atoms bind to the surface of the molecule with a binding energy of about 50 meV. However, it is well-known that regular DFT underestimates the strength of dispersion interactions. Authors in Ref.^43^ used the Grimme dispersion correction to DFT to compute the adhesive energy of the silica-Au interface, which showed that for hydroxylated surfaces, the adhesion consists almost entirely of the dispersion correction. In line with this result, we calculate that the resulting adhesive energy without any corrections is approximately 40 mJ/m${}^2$, whereas the experimental value for hydroxylated surfaces is 360 mJ/m${}^2$ at room temperature^45^. To correct the adhesion predicted by the potential, we deepen the attractive well to yield adhesion energy of 430 mJ/m${}^2$, as shown in Fig. 1. The potential obtained this way also suits better for the intended use at Au-silica surface rather than with isolated Au atoms.

Parameters for the original and adhesive energy-corrected potential are given in Table 1. We note that the experimental value for the adhesive energy (360 mJ/m${}^2$) is measured for hydroxylated glass surfaces. However, silica may contain unpassivated defect sites depending on the experimental conditions. They can form over 2 eV bonds with gold atoms^42^ and increase the adhesive energy^43^. Therefore, even the corrected adhesive energy (430 mJ/m${}^2$) should be considered an underestimation.

**Table 1:** Fitted Morse potential $V(r) = D_e (e^{-2 a (r - r_e) } - 2e^{a (r - r_e})$ parameters for Au–Si, and Au–O interactions.

Parameter	Original potential	Corrected potential
$D_{e,\text {Si-Au}}$	0.3163 eV	0.4260 eV
$a_{\text {Si-Au}}$	1.8536 1/Å	1.8139 1/Å
$r_{e, \text {Si-Au}}$	2.6327 Å	2.5816 Å
$D_{e,\text {O-Au}}$	0.0014 eV	0.0091 eV
$a_{\text {O-Au}}$	0.9082 1/Å	0.8175 1/Å
$r_{e, \text {O-Au}}$	6.3961 Å	5.8094 Å
Adhesive energy	36 mJ/m${}^2$	430 mJ/m${}^2$

Shown is the parameters for the original potential and the one with corrected adhesive energy (see Fig. 1).

### Role of surface adhesion

**Figure 2:**
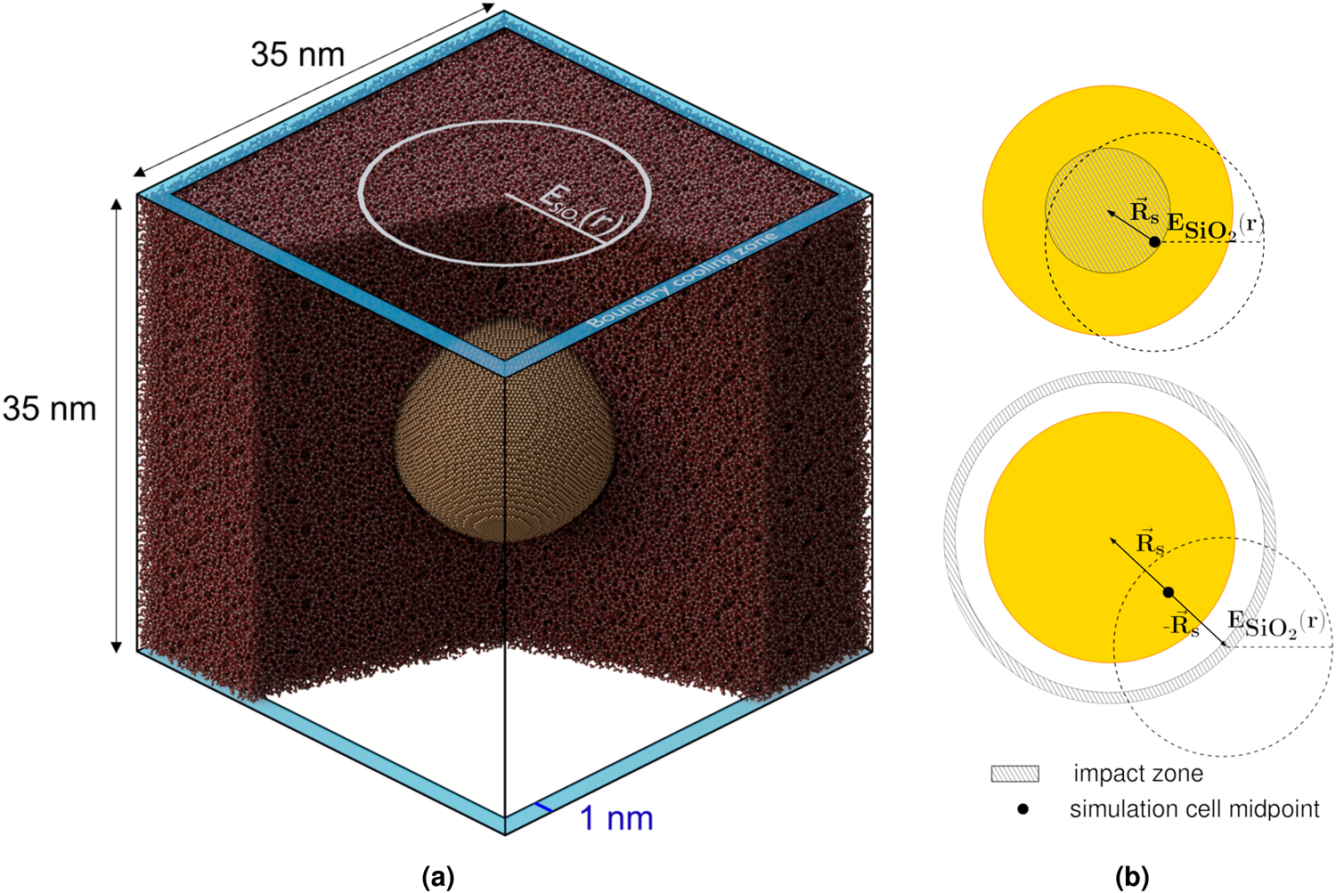
Schematics of the simulation. (**a**) Indicated in the figure are the radial symmetry of the energy deposition profile in silica, cell dimensions, and the width of the boundary cooling volume at the edges of the simulation cell. The radius of the crystalline NP is 8 nm. (**b**) The upper figure shows the region where ions hit the NP. It also shows an example translation, $\vec R_s$, over the periodic boundary to obtain an impact location within the zone. In this case, $|\vec R_s|_{max}$ = 4 nm. The lower figure shows the translation when the ions hit outside the NP. The radial energy deposition profile is also shifted as indicated. The distance from the ion trajectory to the center of the NP is 12 nm, i.e. $|\vec R_s| =$ 6 nm.

To elucidate the role of surface adhesion, we perform two sets of irradiation simulations on spherical NPs with 16 nm diameter using energy depositions estimated for 164 MeV Au ions. The first is based on the potential that is obtained directly from the DFT calculations without any correction (i.e. resulted in the underestimated value of adhesion, $E_{adh}=$ 36 mJ/m${}^2$), while the second includes the adhesion correction ($E_{adh}=$ 430 mJ/m${}^2$). The simulation cell is depicted in Fig. 2a. The path of the ion always intersects the NP, and the impact position is randomly shifted at maximum by half the NP radius between subsequent impacts, as indicated in Fig. 2b. The total duration of simulation of a single impact is 400 ps. Temperature control at the end of every impact to 300 K in the entire cell allowed avoiding the accumulation of energy in the cell.

**Figure 3:**
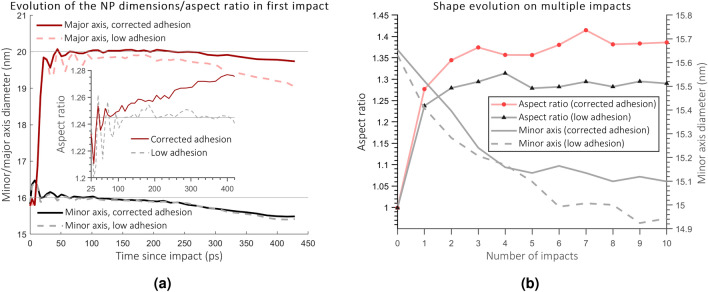
(**a**) NP dimension evolution in the first impact. Solid lines indicate simulations with corrected adhesive energy and dashed without. The inset shows the evolution of the aspect ratio after the ion track in silica has solidified (after about 20–30 ps). It indicates aspect ratio growth during the cooling in the simulations with corrected adhesive energy, whereas the low adhesion NP cools and shrinks isotropically without prominent changes in aspect ratio. (**b**) The evolution of the aspect ratio and the minor axis size on multiple impacts.

We begin the analysis by examining the first impact in the irradiation series. In our irradiation simulations, both types of simulations indicate aspect ratio growth as shown in Fig. 3a and described previously^9,28,39^. During the first 20 ps, molten, high-pressure gold pushes into the underdense ion track in silica. The initial part of the simulation, when most of the aspect ratio growth occurs, behaves identically in both simulations. However, the total aspect ratio growth is higher in the simulation with higher adhesion, and differences occur after 20 ps. At this time, the molten ion track in silica solidifies, while the NP still remains molten. The NP and the ion track in silica cool down due to heat dissipation from the track region to bulk, but the cooling is significantly faster in silica than in the NP due to weak heat conduction through NP/silica interface. After the freezing of the ion track, no more material can flow to the ion track. Therefore, no further increment in the major axis size of NP is possible. However, the aspect ratio growth continues in the simulation with the high adhesive energy even after 20 ps.

Since the minor axis evolves identically in both simulations (the minor axes in both cases shrink during the cooling phase), the behavior of the material along the major axis explains the difference in aspect ratio development during the impact and right after it, see Fig. 3a. The NP with the low adhesion detaches almost completely from the ion track in silica and shrinks during the cooling phase almost isotropically. Such behavior will not lead to further changes in the aspect ratio during cooling, and small voids form instead above and beneath the particle. However, the NP with the corrected adhesion to the ion track resists the formation of these voids, and the major axis does not shrink in size as much as with the low adhesion. As a result, the aspect ratio keeps increasing as the NP cools down. This observation demonstrates that shape change can also occur after the ion track has solidified. It is also worth noting that no width loss occurs during the initial part of the simulation. When the modified NP cools down, its elongated shape inevitably leads to further loss in its width.

### Effect of multiple impacts

**Figure 4:**
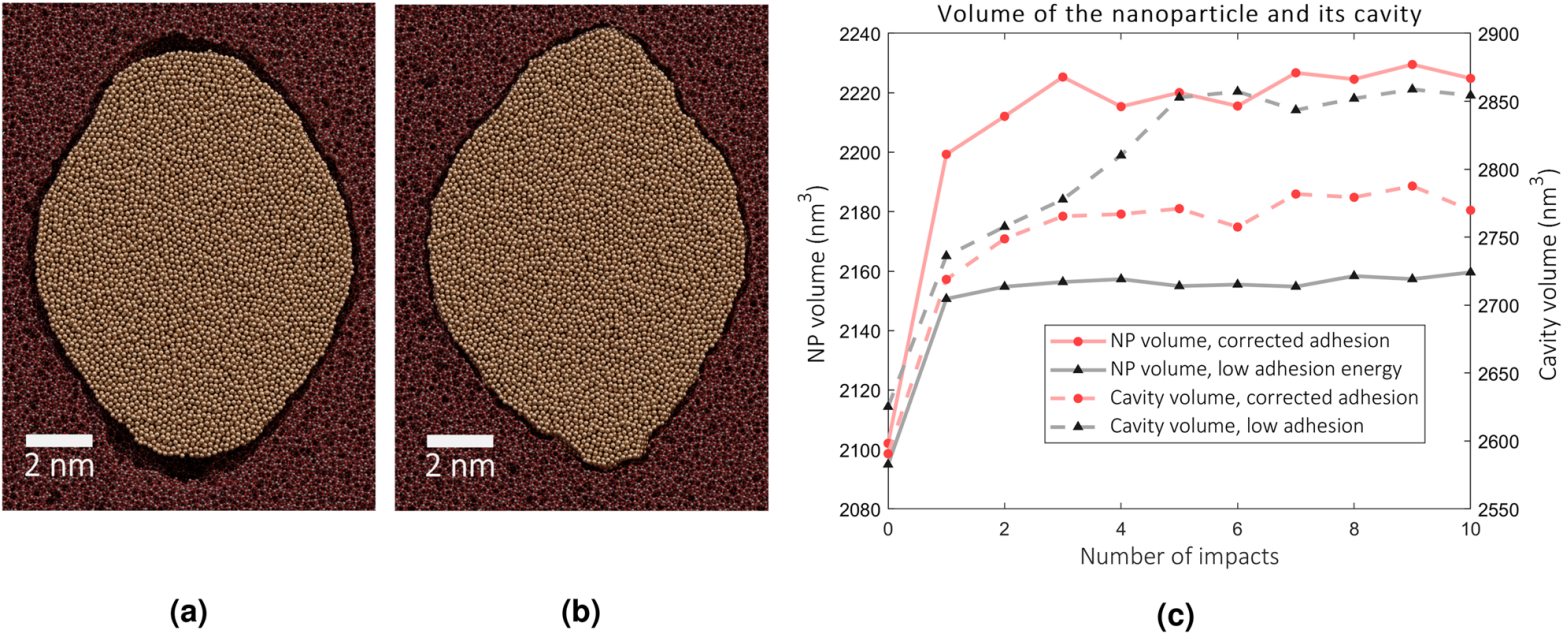
(**a**) Snapshot of the cell after the 10th impact at 300 K for the simulation with low adhesion (cell cut in half). It shows the amorphous structure of the NP and an empty region around it. Larger voids can be seen on top and below the NP. (**b**) Shown is the same for the simulation with corrected and stronger adhesion. No empty regions are visible around the NP. (**c**) The volume of the NP and the cavity of the NP (the void that would result by removing all Au atoms from the system) at 300K.

Similar differences persist in the simulations also on later impacts. After ten impacts, the aspect ratio has grown to the values of 1.3 and 1.4 for the simulations with the low and corrected adhesion potential, respectively, as seen in Fig 3b. This confirms that the adhesion affects the elongation rate. Both particles continue to lose width and change shape on the first impacts but reach a state by the tenth impact in which no further, significant changes occur. The voids above and beneath the NP in the simulation with low adhesion are visible in the snapshot presented in Fig. 4a. Moreover, around the whole NP one can see a thin empty layer as well. No such layer is visible in the simulation with corrected adhesion, shown in Fig. 4b. Instead, the particle has a uniform, seamless interface with silica.

In Fig. 4c, we show the evolution of the NP volume and the NP cavity (i.e. the void in silica wherein the NP resides) obtained in both simulations with the low and the corrected adhesion. The figure shows that the NP volume in both simulations increases with respect to the original state in the crystalline FCC configuration, although the increase of the NP volume with stronger adhesion is larger. In the latter case we also observe that the volumes of the NP and its cavity evolve similarly (note the different scale for the NP and the cavity volumes). With low adhesion, the cavity grows faster, while the volume of the NP saturates after a few impacts and does not follow the same trend as the cavity growth.

In order to reach the experimental fluences (for example, 2 $\times$ 10${}^{14}$ ions/cm${}^2$ in Ref.^9^), which result in transformation of initially spherical NPs into nanorods, the NP must experience at least a hundred of ion impacts in our simulations. The impacts near the edges of the NP are not expected to raise the temperature above the melting point^30^. On the other hand, the elongation rate depends on the temperature of the cluster and its melting^39^. Since we simulate the ion impacts only at the central part of the NP, the latter should elongate strongly with fewer impacts. However, both simulation series indicate saturation of the aspect ratio growth, as shown in Fig. 3b. To confirm the saturation in the case of stronger adhesion, we further extend the total number of impacts to 14. No further net change occurs (see SI Fig. 1). The saturation was also previously observed in Refs.^9,39^.

We argued in Ref.^9^ that the small lateral size of ion tracks imposes fast cooling dynamics. The NP that has been thermally expanded after an ion impact, freezes in the expanded volume in an amorphous-like structure. This leads to smaller subsequent relative expansion of the NP volume limiting further shape change. However, long time intervals between the experimental subsequent impacts on the same NP suggest that the NP may have sufficient time to recrystallize before the next ion hits it, triggering the thermal expansion process again. Hence, a “recrystallization” step - the NP after each impact was rebuilt in a crystalline structure conserving the shape and the number of atoms - was introduced. This step is a crude approximation of a long time recrystallization process and requires verification. On the other hand, the authors in Ref.^28^ obtained a higher aspect ratio using a similar simulation setup without the recrystallization procedure. The main difference to the simulations presented here is the notably higher temperature to which NP was heated (compare 9000 K^28^ vs. 2000 K^9^). Much higher temperature causes stronger expansion of NP enabling continuous elongation. However, the lateral dimension of NP in this study did not shrink, indicating that NP remained in a strongly-expanded state even at the end of the simulation. Elongation by expansion only will lead to a metastable state with high negative pressure within it, which can cause the NP to collapse. While some volume growth could occur due to amorphization and atom mixing, the elongated NPs have been shown to have crystalline structure^4^. Indeed, we observe a negligible amount of mixing of silicon and oxygen atoms into the NP in our MD simulations that may be more significant in experiments under much higher fluence.

There is still an uncertainty with respect to temperature that develops within the NP due to an ion impact. Hence, the elongation indeed might be driven by very high temperatures that may develop within the NP as it was shown in Ref.^28^ To study this, we ran additional set of simulations, where the temperature within NP was set to 3000 K during impacts. This simulation confirmed that the NP initially elongated more but eventually collapsed during cooling so that all aspect ratio growth is lost (see SI Fig. 2).

### Long time scale evolution

To shed light on phase transition in the NP that may take place between the subsequent impacts, we study a scenario in which the NP was able to relax for a longer time between impacts than those which were used in previous MD simulations. We expect that the recrystallization provides a mechanism by which the NPs can shrink in volume to be able to elongate during the next impact^9^. However, we note this mechanism reduces the volume of the NP only and is not able to reduce the volume of the cavity, if the adhesion model predicts weak interactions. Empty region around the NP is visible in Fig. 4a and can be verified in Fig. 4c. Therefore, even if the NP reverts to the original volume, it might only expand to the enlargened cavity during the next impact without a further net change in aspect ratio. The forced recrystallization process introduced in Ref.^9^ bypasses this issue since the size of the cavity is automatically adjusted to the size of the NP. In the high-adhesion simulations, the cavity grows slower than in the low-adhesion ones (see Fig. 4c), but the NP that is coupled with the cavity and, hence, prevents it from growing faster, remains in an expanded state. We now set the long-timescale simulation to investigate whether the relaxation process can decrease the volumes of the NP and the cavity coupled by strong adhesion so that the elongation can proceed as explained previously. Due to the high computational costs of the simulation and improved realism, we simulated the long-term processes only for the NP with corrected adhesion.

**Figure 5:**
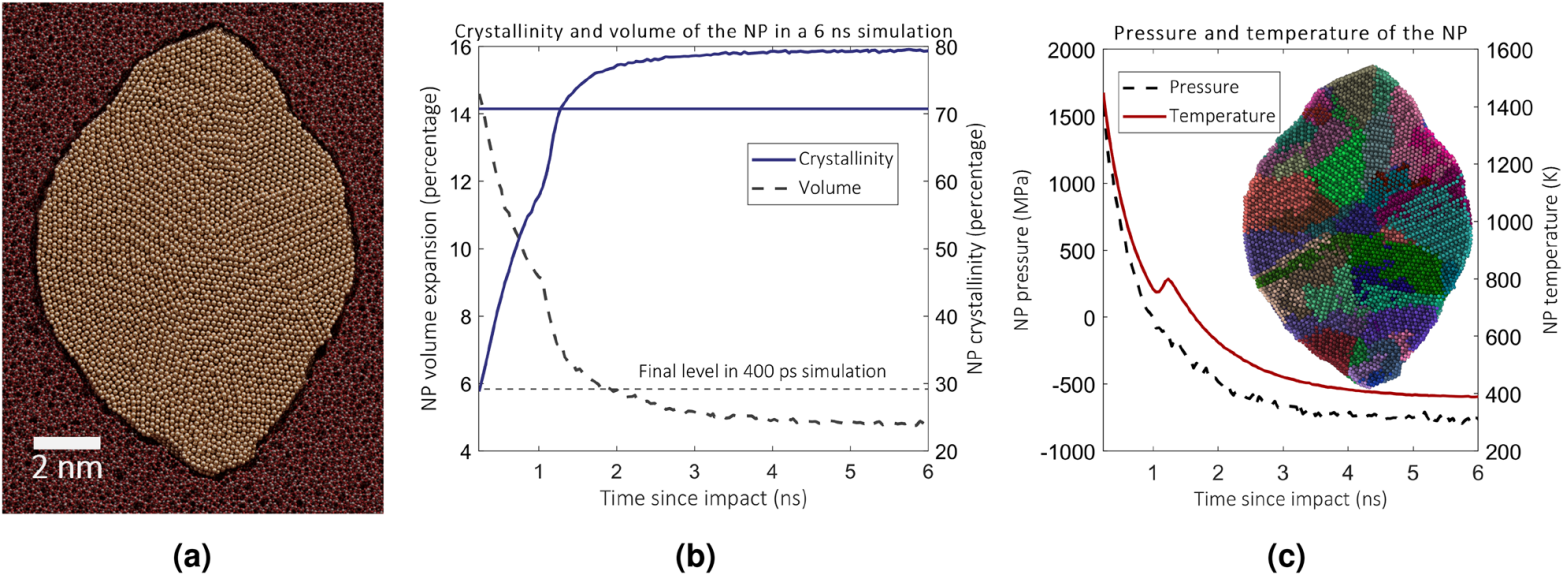
(**a**) Snapshot of the simulation cell after the 6 ns simulation. The cell is cut in half. A crystalline grain is clearly visible in the upper-right corner of the NP. Rest of the grain do in the (**b**) Crystal content and the volume of the NP as a function of time in the long MD simulation. Shown by the horizontal lines are the corresponding levels in the simulations without the long MD simulation between impacts. c) Shown is the temperature and the pressure of the NP. The inset shows colored grains that are identified using the grain segmentation tool in OVITO^46^.

During the regular simulation cycle, the NP evolves for 230 ps after the impact with boundary conditions that mimic heat conduction to the bulk. Another 200 ps is reserved to cooling the NP to 300 K and pressure relaxation. Now instead of the regular 200 ps run, we let the NP cool down with only boundary cooling for six ns after each 10th impact. The crystallinity, volume, temperature and pressure of the NP during the simulation are shown in Fig. 5. Figure 5a shows a snapshot of the simulation at the end. It is evident that crystalline grains form and, as argued previously, the shape of the particle does not change. Figure 5b shows that the particle reaches about 80% crystallinity, but the NP is still about 4% over its original volume at the end of the simulation. Figure 5c shows the evolution of the temperature and pressure during the relaxation. Initially, a large overpressure (> 1.5 GPa) forms in the NP in line with previous arguments. However, as the particle cools down, a significant underpressure (-0.8 GPa) develops in the cluster. Even after cooling down, the volume of the NP is larger than that of the NP with low adhesion in the amorphous state. Moreover, the final cavity size does not change compared to the shorter simulation. Hence we conclude that another mechanism is required to decrease the volumes of the NP and its cavity. Furthermore, the adhesion between the NP and the cavity causes considerable underpressure in the NP, which will decrease the initial pressure on subsequent impacts.

**Figure 6:**
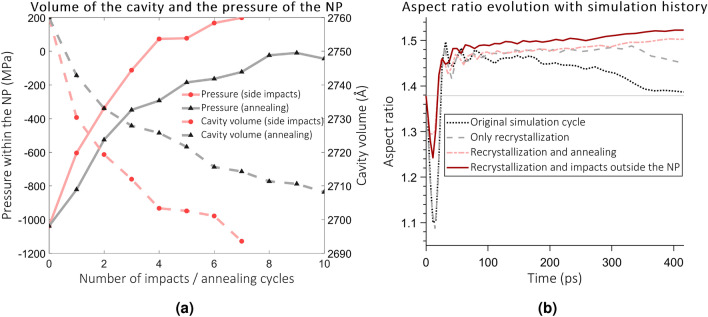
(**a**) The pressure and the cavity volume (void that would result by removing Au atoms) during impacts nearby. Also shown are the same results from an alternative annealing treatment. (**b**) Comparison of aspect ratio growth on the 11th impact using simulation cells with different histories.

Another process that may play a role but thus far has not been considered in MD simulations is the ion impacts in the vicinity of the embedded NP, but not directly on it. A realistic simulation of SHI irradiation of an embedded NP will require simulation of subsequent ion tracks in random locations of the simulation cell, with no specific regard to the location of the NP itself. However, only about 16% of the ions hit the NP, which affects the efficiency and computational costs of the simulations. To study the effects of the side impacts, we use the simulation cell with the elongated Au NP, which was simulated for additional six ns to allow for its natural recrystallization. In this series of simulations, the ions hit in a circular region around the NP that has a radius 2 nm greater than that of the NP, as illustrated in Fig. 2b. The volume of the NP and the pressure within it after each impact are shown in Fig 6a. After 7 impacts, the negative pressure within the NP has decreased and the width of the NP has reduced. The simulation cell shows characteristics of the compactification preceding the ion hammering effect: the density of silica has increased by about 10%. The simulations show that the cavity size decreases by the nearby impacts.

However, irradiating the cell in this manner is still computationally prohibitively expensive. As an alternative method to mimic the evolution of the cell under thermal spikes, we anneal the simulation cells and control the pressure. In the annealing cycle, the temperature of the cell is increased linearly to the target temperature and cooled back to the room temperature. The heating rate (and cooling, 120 K/ps) and the target temperature and pressure (3500 K, 0 GPa) are chosen so that there is not enough time for melting to occur, but atoms gain enough kinetic energy to find new equilibrium positions. As seen in Fig. 6a, about 8 annealing cycles remove the negative pressure from the NP. We note during this process, the ion track structure (density changes induced by the ion) in silica is preserved, and the median displacement of atoms during a single annealing cycle is only 0.4  Å. The median after nine annealing cycles is 3.6 Å.

### Effect of the long timescale evolution upon the subsequent impacts

**Figure 7:**
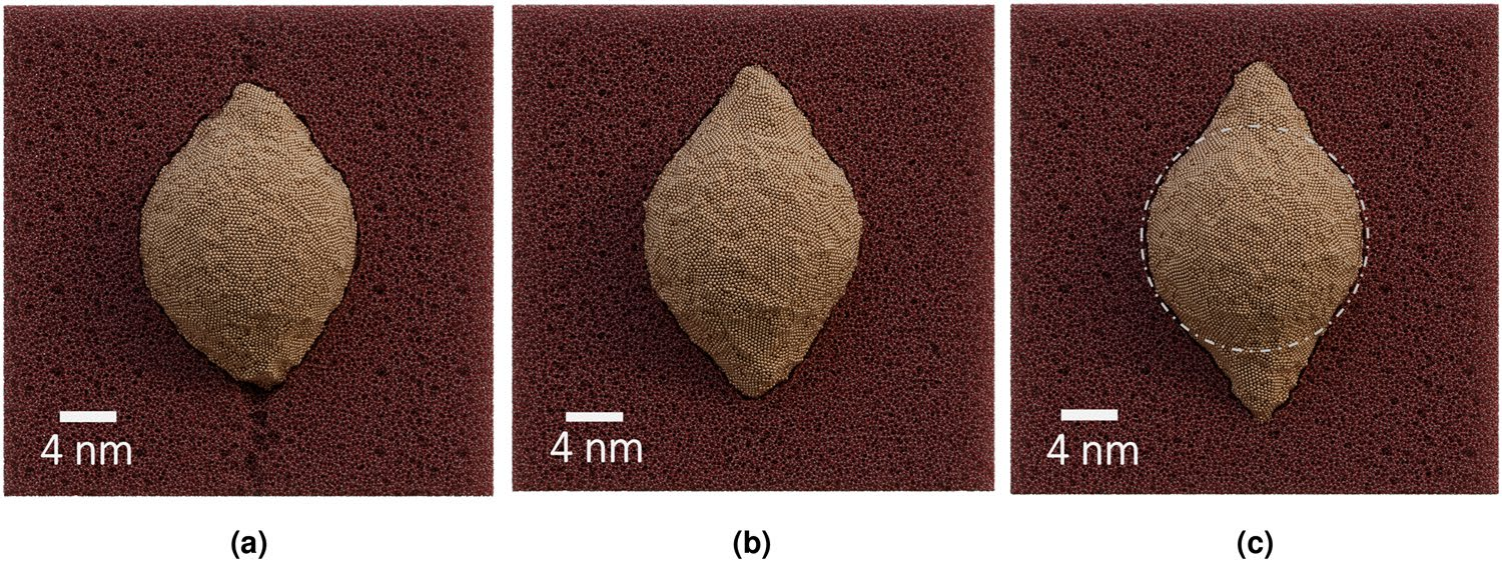
(**a**) Shown is the NP after the 11th impact using the original simulation cycle (the NP is not cut in half) (**b**) As previous, but with 6 ns simulation and impacts outside the NP (**c**) NP after 12th impact using the alternative the annealing method on impacts 10 and 11. Indicated by the white dashed line is the shape of the NP at the start of the simulation.

Finally, we consider how the relaxation processes affect the evolution of the aspect ratio upon subsequent impacts. A simulation is performed in cells with different histories so that the ion hits the central axis of the NP. Results of this comparison can be seen in Figs. 6b and 7. Simulation with the original cycle shows negligible aspect ratio growth (see Figs. 6b and 7a). The six ns recrystallization simulation increases the aspect ratio growth compared to the original simulation cycle only modestly. A clear difference occurs when the long recrystallization simulation is continued with the impacts in the vicinity of the NP (Figs. 6b, 7b) or when using the thermal annealing approach instead. We have also performed a second subsequent impact with the annealing process. In this test, the NP is relaxed again with a six ns simulation for recrystallization and the cell again annealed. A prominent aspect ratio growth occurs again from the initial value of 1.5 to a final aspect ratio of 1.64 (note that the corresponding aspect ratio is 1.38 in the original cycle). The NP is shown in Fig. 7c. Therefore, we conclude that the saturation is an artificial effect not manifesting in a more realistic simulation setting. Moreover, the figure shows that the NP has lost width, although the decrease is only $\sim$1 nm due to the small number of impacts. A similar diagram for the snapshots presented in earlier works^11,39^ indicates expansion only.

### Experimental characterization of the nanoparticle and the metal-matrix interface

**Figure 8:**
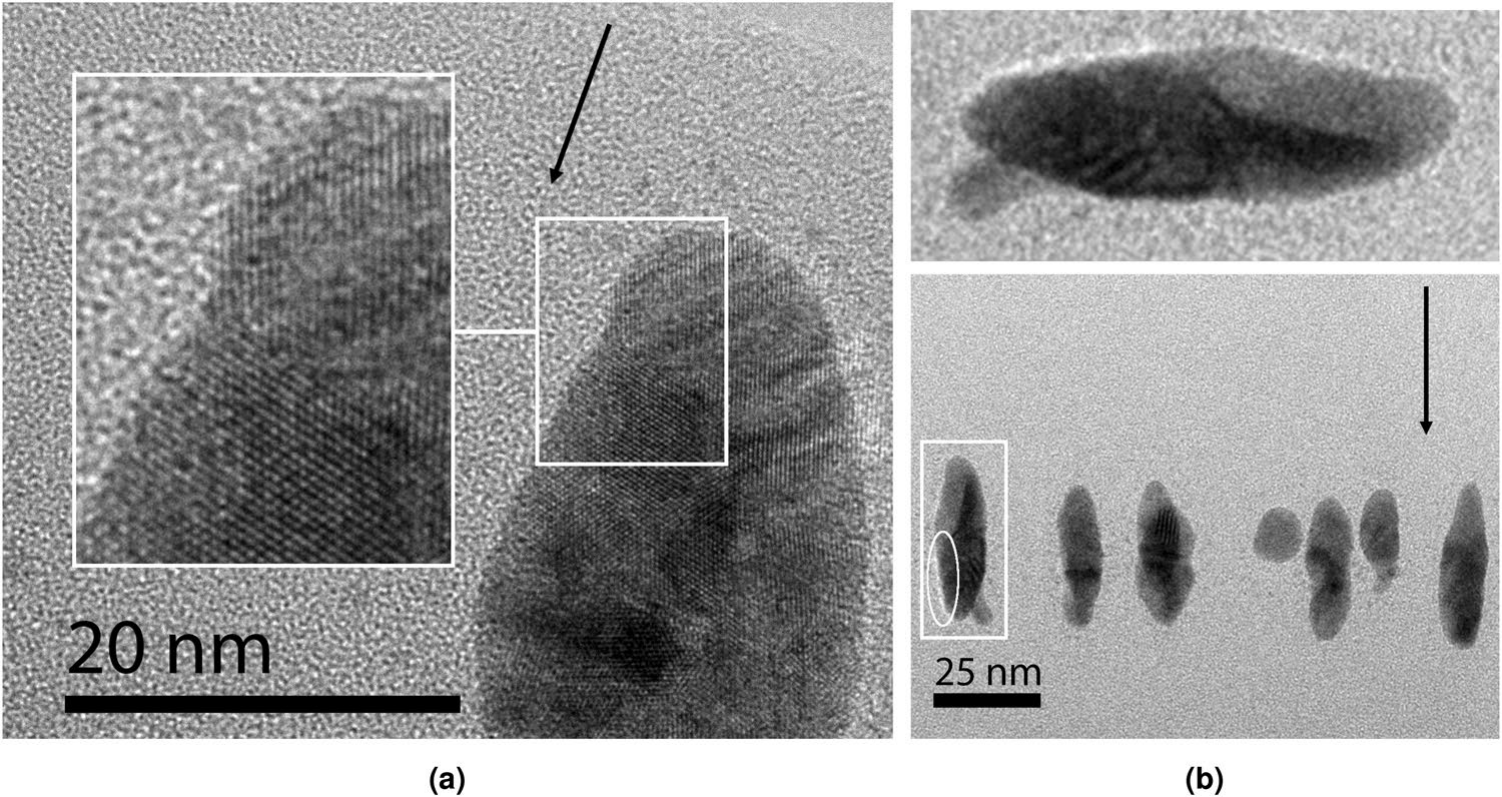
Cross-section HR-TEM micrographs of spherical Au NPs irradiated using 185 MeV Au ions to a fluence of $3 \times 10^{13}$ cm${}^{-2}$. Initially, the particle diameters are about 18 nm, and the fluence corresponds to around 100 impacts within the same diameter. The irradiation direction is indicated by the black arrow. (**a**) High-resolution image (magnification is $\times$2 000 000) showing the crystalline structure of the nanoparticle and its interface with silica. Two distinct crystalline grains with different orientations are visible from the highlighted area. (**b**) Elongated NPs showing void-free interfaces between silica and Au except for the particle highlighted by the white rectangle. The white region on one side of the particle, delimited by the ellipse, is indicative of a void or a low-density region that conforms to the shape of the NP. Shown on the top is the highlighted particle magnified. The image was taken with a $\times$300 000 magnification to observe the elongation of the NPs in a single layer.

Au NPs embedded in silica with a mean diameter of about 18 nm were irradiated with 185 MeV Au ions and imaged using cross-section transmission electron microscopy to characterize the structure of the elongated NPs and their interface with silica. Judging from the flux, the typical time between the impacts to the NP is about 30 seconds, so the recrystallization process observed in the simulations must have taken place. The experimental fluence corresponds to about 100 impacts to the NP. HR-TEM micrographs are shown in Fig. 8. As in the simulations, the NP is polycrystalline but the grain size is larger. Grains bigger than 10 nm in width are visible from the micrograph shown in Fig. 8a, while in the simulations, the maximal grain width is about half of that. This discrepancy is likely due the different timescales for recrystallization. Inspection of the interface structure reveals that silica forms a closely connected interface with the NP without apparent gaps. Like in the simulations, the interface is irregular in shape. However, examination of several NPs reveals that sometimes a lower-density region or void can form next to the particle. Such a feature can be seen at the bottom left of the NP in Fig. 8b, highlighted by the ellipse. Interestingly, the shape of the void conforms to the shape of the NP. Therefore, it might result only from the volume loss that follows the enlarged-volume molten state after impact. It is plausible that a partial rupture from silica can occur due to the negative pressure especially in large NPs. Gaps surrounding the whole NP, as seen in the simulated figure without adhesive correction in Fig. 4a, were not observed. We also point out that, according to our theoretical predictions, some of the NPs may have a positive strain. While the strain cannot be estimated currently from the micrographs with sufficient accuracy, future experiments should enable such analysis.

## Conclusions

Our MD simulations have shown that the elongation process involves many processes that take place during SHI irradiation. The driving force for NP elongation is the material flow caused by the thermal expansion of the molten and strongly heated NP into the underdense and molten core of the ion tracks in the matrix. After solidification, the matrix supports the modified NP shape, and there is enough time for recrystallization of the metal lattice between each impact. During the recrystallization, adhesion between the NP and the matrix causes a negative pressure to develop within the NP. Only the ions that hit in vicinity of the NP reduce the negative pressure within it. Hence, to explain the elongation process, it is insufficient to consider only the ion impacts directly on the NP, even if the location of these impacts is randomized. These insights elucidate seemingly incompatible experimental observations: elongation is induced by individual impacts on NPs, while the processes developed in the embedding matrix under irradiation play a crucial role on the elongation as well.

Experimental characterization of the elongated NPs and their interfaces reveals a structure consistent with the simulations. HR-TEM images show a polycrystalline NP structure and a seamless interface with silica. The micrographs also show that small voids or “gaps”, possibly remnants of the high-volume state following an impact, can sometimes form at the interface. Their formation might be due to a rupture mechanism to relieve the negative pressure.

We have demonstrated the process to be more complex than thought earlier, and a lot remains to be explored. Despite already a long history of experimental research, only a handful of papers address the shape transformation problem theoretically. We hope that our work inspires and paves the way for further analysis. The insights presented here are a step toward accurate simulations and controlled shaping of the NPs experimentally.

## Methods

### Simulation procedures

#### Interatomic potentials

Si-O, Si-Si, and O-O interactions were described by the Munetoh potential^47^, which is of Tersoff-type. Au-Au interactions were depicted by an EAM-potential by Foiles et al.^48^. For Au-Si, Au-O interactions, we developed a pair potential fitted to first principles calculation made with the Gaussian 16^49^ software.

We used the DFT method with the Becke 3-parameter Lee-Yang-Parr (B3LYP) hybrid functional and a mixed, localized basis set. On Si and O atoms, Pople-style basis set augmented with diffuse functions (6-311++G(d,p)) was used, whereas for the gold atom, Los Alamos National Laboratory 2-double-z (LANL2DZ) basis set was used. Similar setup was used, for example, by Del Vitto et al.^42^ and Lu et al.^50^

#### Details of the Au-silica potential computations

We first relaxed the structure so that the gold atom lies outside of the molecule, at the symmetry axis (x-axis). Potential energy surface scan was then performed along the symmetry axis as shown in Fig. 1. The total energy at x = 15 nm was chosen as the zero level energy, and subtracted from the DFT results. Origin was chosen so that x = 0 nm means gold is in the middle of the molecule. To express the total energy from the DFT computation as individual pair contributions from Morse potentials, the total energy was evaluated as

1 $$\begin{aligned} \begin{aligned} E_{\text {Morse}}(x) = \sum _{i=\text {Si}_1...\text {Si}_8 - \text {Au}} D_{e,\text {Si-Au}} ( e^{-2a_{\text {Si-Au}}(r_i(x) - r_{e,\text {Si-Au}})} - 2e^{-a_{\text {Si-Au}}(r_i(x) - r_{e, \text {Si-Au}})} ) \\ +\sum _{i=\text {O}_1...\text {O}_{12} - \text {Au}} D_{e,\text {Si-O}} ( e^{-2a_{\text {Si-O}}(r_i(x) - r_{e,\text {Si-O}})} - 2e^{-a_{\text {Si-O}}(r_i(x) - r_{e, \text {Si-O}})} ) \end{aligned} \end{aligned}$$

where the summations go over all gold atom pairs in the system, and $r_i(x)$ is the radius of an individual bond when the gold atom lies at *x*. $D_e$, *a* and $r_e$ pairs for Au-Si and Au-O are the well depths, bond widths and equilibrium distances for each type of interaction. These six parameters were optimized by minimizing the squared difference between the energies obtained from DFT potential surface energy scan and those evaluated using Eq. (1) at the scan locations (shown by the dots in Fig. 1). The Jacobian matrix with respect to the parameters was formed using analytic differentiation, and the mimization was performed using a Gauss-Newton algorithm. The resulting parameters are given in Table 1 and a comparison between the fitted and DFT potential energy in Fig. 1.

We also computed corrections to the potential based on the experimental adhesive energy. To compute the adhesion energy predicted by the fitted potential, we prepared a spherical Au NP in cubic a silica simulation cell as described in section ’Simulation procedures”. The NP radius was 8 nm. Basing the computations on the the relaxed room temperature system as the (300 K, 0 GPA), we estimated the adhesive energy as (following Ref.^43^)

2 $$\begin{aligned} E_{\text {adh}} = \frac{E_{\text {Au}} + E_{\text {SiO}{}_2} - E_{\text {Au/SiO}{}_2}}{4 \pi r^2} \end{aligned}$$

where *r* is the NP radius, $E_{\text {Au}}$ and $E_{\text {SiO}{}_2}$ refer to the total energies of the gold nanoparticle and the cubic simulation box separately, and $\text {Au/SiO}_2$ to the energy of the composite system.

To fit the adhesive energy, we multiplied the potential energy values given by the original fit on the right hand side of the potential well minimum with different scaling factors (2,4,6,10). The potential energy values far from the minimum were kept unchanged (values in the range $x \lessapprox 4$ Å in Fig. 1), and the remaining ones removed. A new fit to the resulting potential energy values for all the scaling factors was performed. The resulting new potentials were used to construct the NP in silica system from the beginning. It was observed that using value of four as the scaling factor gave the closest adhesive energy value to the experimental one.

All MD simulations are performed using the LAMMPS^51^ software package (http://lammps.sandia.gov).

#### Initial cell for impact simulations

A smaller cubic simulation cell with a side length of 5.8 nm was obtained with the WWW-method as described elsewhere^52^. It was then multiplied six times for a larger silica cell (35 nm × 35 nm × 35 nm) and relaxed with the Munetoh potential. A sphere with a diameter d = 16 nm was cut from FCC Au, compressed by 1%, and inserted into a spherical cavity in the silica cell, which was cut slightly larger (d = 16.2 nm). Next, the system was equilibrated for 100 ps towards 0 GPa/300 K using a Nose-Hoover thermostat and a 0.8 fs time step (thermostat time constants were 80 fs for temperature and 800 fs for pressure). The purpose of this procedure is to make the initial cell stress-free. Moreover, the compression forces the NP to interact with silica without low interatomic separations in the initial configuration that might result in atoms with high velocity near the surface.

#### Impact simulation and the main simulation cycle

Schematics of the simulation cell configuration for impact simulation is given in Fig. 2. To model the rapid heating of atoms following a SHI impact, random velocity vectors are added instantaneously so that the kinetic energy increase corresponds to that estimated from the two-temperature model for silica. For gold, we have tried both instantaneous and heating the NP to 2000 K in 5 ps duration to account for the slower electron-phonon interactions. We chose use the latter approach for the main simulation cycle, as it increases the realism according to the two-temperature model. The value of 2000 K is chosen on qualitative grounds, but in line with earlier estimates using the two-temperature model with the Au NP-silica system^6,30^ and similar ions. It is enough to melt gold (melting point with the EAM potential is 1110 ± 20 K^9^) but enough not to vaporize it. When the ion does not hit the NP (see Fig. 2b), a temperature of 600 K is used instead, which is in line with earlier estimates^30^. The instantaneous radial deposition profile in silica is based on 164 MeV Au ion and the as same used in Refs.^9,39^ and is shown in SI Fig. 3 for completeness.

The simulation cell is cooled at four boundaries parallel to the ion direction to dampen pressure waves and to mimic heat conduction to bulk. The width of the boundary cooling region is 1 nm and the cooling is performed using a Berendsen thermostat with a rapid time constant (10 fs) towards 300 K. Once the heating of Au is over, simulation continues with the boundary thermostat still turned on for 230 ps. This stage is followed by a linear pressure/temperature relaxation to 0 GPa and 300 K with a total duration of 200 ps using a Berendsen barostat and thermostat. The thermostat is applied separately to all silica and Au atoms to ensure that both systems are at 300 K before the next impact (and not only the average of the two). After this relaxation, shifting over periodic boundaries is performed as explained in the main text, and the simulation cycle is started from the beginning for more impacts to the cluster.

#### Nanoparticle characterisation

The dimension and the aspect ratio were computed using an average position of 100 furthest atoms in each dimension. The volume of the NP was determined using the coordinates of gold atoms and the convex hull volume determination in MATLAB (The MathWorks Inc., Natick, MA). For quantifying the size of the cavity, Voronoi analysis was used to determine the first silica neighbors of gold atoms. We determined the convex hull volume of these first neighbors, and computed its volume. To characterize the crystal content in the NP, we used the Ackland-Jones analysis^53^ as implemented in the OVITO software^46^. The percentage of crystalline content was computed as the ratio of atoms in any crystal structure (FCC, HCP, BCC, ICO) over unidentified atoms.

### Experimental procedures

The synthesis of Au NPs was carried out by deposition of a thin silica layer with a thickness of 500 nm on a c-Si (100) wafer by plasma enhanced chemical vapor deposition (PECVD), followed by the deposition a 5-nm Au layer by thermal evaporation. The Au layer was then covered by a second silica layer with a thickness of 500 nm. Then, the sample was annealed in a rapid thermal annealing (RTA) process for 2 min at 1000 $^\circ$C in a N${}_2$ atmosphere, resulting in Au NPs with a mean diameter of 17.8 ± 5.4 nm^54^.

The semi-spherical Au NPs were irradiated with 185 MeV Au${}^{13+}$ to fluences up to 3 $\times$ $10^{13}$ cm${}^{-2}$ to promote the shape transformation. The corresponding energy-loss rate was estimated using the SRIM 2010 code^55^. With a total layer thickness of  1 μm, the electronic energy-loss rate varies within 1% and thus can be estimated by the surface values, resulting in a value of 16.3 keV/nm for silica and 50.0 keV/nm for Au. The irradiations took place at the Heavy Ion Accelerator Facility (HIAF) at the Research School of Physics at Australian National University (ANU). The peak current was 4 nA and the beam is scanned across a 6.1 mm $\times$ 3.2 mm area, aiming to cover the area as homogeneously as possible. This corresponds to a flux of about 9.8 $\times$ $10^9$ ions / cm${}^2$s. More details on the irradiation procedures can be found in Ref.^56^

The elongated NPs were characterised by cross-section HR-TEM with a JEOL 2100F microscope operated at 200 kV. The specimen preparation was carried out with a precision ion polishing system (PIPS) GATAN model 961 at − 165 $^\circ$C to minimize unwanted warming up of the sample after mechanically dimpling the sample to a thickness of 10 μm.

## Supplementary Information

Supplementary Information.

## Data Availability

Example simulation scripts for LAMMPS are available from https://github.com/aaleino/NP_shape_modification. Other data supporting the findings from the study are available from the corresponding author upon reasonable request.
