# Supplementary material for: Insights into nanoparticle shape transformation by energetic ions
Source: Sci Rep. 2023 Apr 18;13:6354. doi: 10.1038/s41598-023-33152-9 (PMC10113260; doi:10.1038/s41598-023-33152-9)
Supplement: Supplementary file 1 — Supplementary Information. [file 41598_2023_33152_MOESM1_ESM.pdf]

Supplementary Material: Insights into nanoparticle shape transformation by energetic ions

Aleksi A. Leino[1,*], Ville E. Jantunen[1], Pablo Mota-Santiago[2,3], Patrick Kluth[3], and Flyura Djurabekova[1]

[1]Department of Physics, University of Helsinki, Helsinki, P.O. Box 43, FI-00014, Finland

[2]MAX IV Laboratory, Lund University, Lund, P.O. Box 118, SE-22100, Sweden

[3]Department of Materials Physics, Research School of Physics, Australian National University, Canberra ACT 2601, Australia

[*]aleksi.leino@helsinki.fi

1 Saturation of growth using more impacts

Fig. 3b in the manuscript shows the evolution of the aspect ratio up to ten impacts. It might appear from the graph that the NP with corrected adhesion is still slowly increasing in aspect ratio. To show that this is not the case, the evolution during four more impacts is presented in SI Fig. 1.

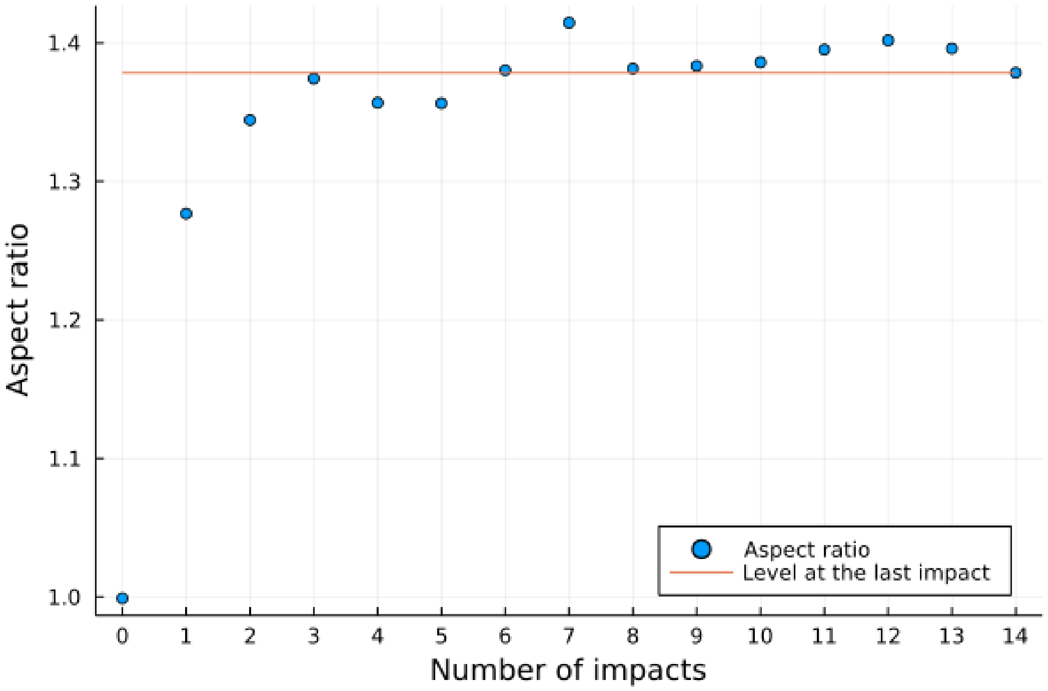

SI Fig. 1. The evolution of the aspect ratio for 14 impacts with the corrected adhesion.

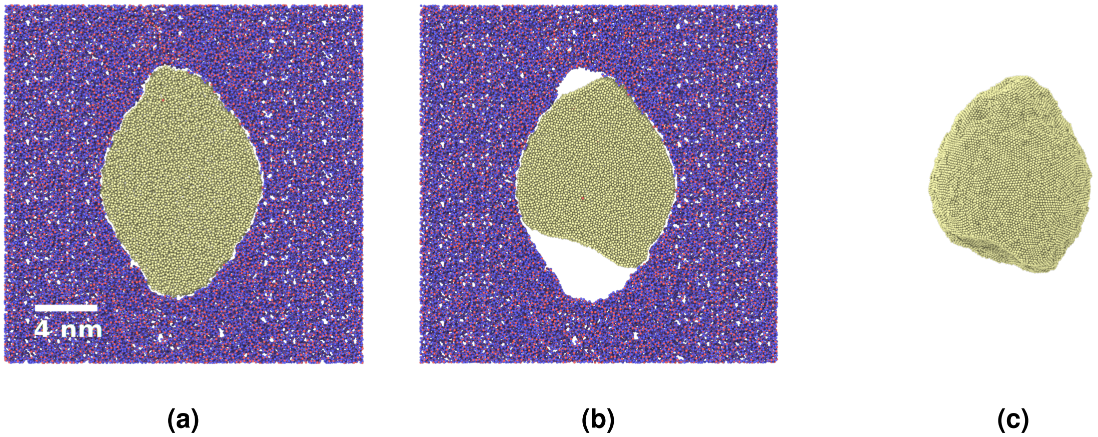

SI Fig. 2. Collapse of the nanoparticle at 3000 K. a) Cross sectional snapshot of the simulation cell before the collapse b) after the collapse c) All atoms of the NP after the collapse.

2 Collapse of the nanoparticles at elevated temperatures

In the section ”Effect of multiple impacts,” it is mentioned that the nanoparticles collapse when a heating temperature of 3000 K is used instead of 2000 K. Also, in this test, a heating time of 2.5 ps was used over the 5 ps time that was use elsewhere. The collapse occurred during the 8th impact on the NP. Snapshots before and after are shown in SI Fig. 2. The collapse occurs during the cooling of the cell and can be explained by the high volume expansion NPs. Since the expanded volume of the NP is higher, the negative pressure accumulating in the cluster, which causes the collapse.

3 Energy deposition profile in silica

The energy deposition to silica was the same as used in Refs.[1–3] and shown in SI Fig. 3. This profile is based on the so called inelastic thermal spike model and was provided to us by M. Toulemonde. It had previously given a track diameter in agreement with experiments[2,3]. The parameters used in the computations are given in Ref.[4] The profile results from solving a coupled set of heat diffusion equations. These equations can be used to compute how much the lattice temperature increases as a function of time, which can be translated in the units of eV/atom. The profile is extracted at a time instance when the lattice temperature has reached maximum. This occurs shortly ($\sim$ 100 fs) after the impact. The inelastic thermal spike calculation was computed for a beam with energy 1.1 MeV/u, and rescaled by 4/5 to obtain the total electronic stopping power of 14.4 keV/nm (close to the SRIM value of 16.5 keV/nm for 185

MeV Au ion[5]). The rescaling was done since the profile was originally used as part of a five-point sweep that studied how much the total energy deposition affects the qualitative features of ion tracks (see Fig. 3 in Ref.[3]). As discussed in Refs.[3,6], the resulting profiles from the inelastic thermal spike model has been multiplied by a factor of two. This discrepancy was attributed to the inability of the potential (there, Watanabe-Samela potential[6]) to describe the melting point accurately. We note that for the purposes of the current study, this profile is only used in qualitative manner. However, using the same profile (i.e. multiplied by 2) with the Munetoh potential (used in the current work) gives quantitatively similar features for the ion track (i.e. the ion track with similar radius and underdense core). Since these are dependent on the melting point, the similarity indicates that the Munetoh potential also overestimates the melting point.

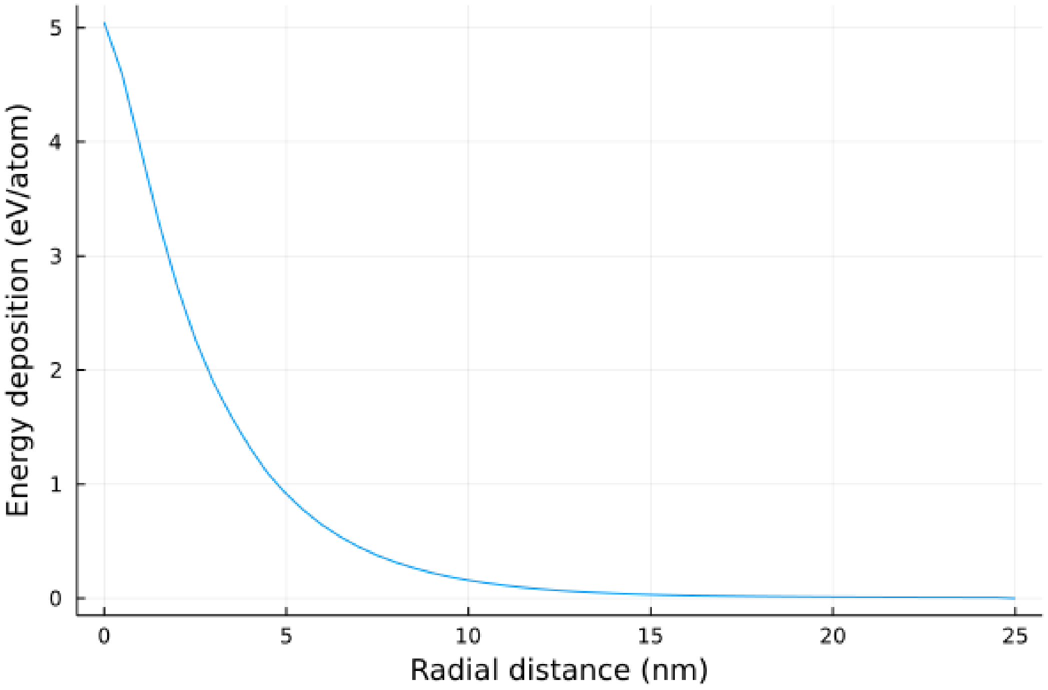

SI Fig. 3. Energy deposition to silica as a function of radius.

References

1. Leino, A. A., Pakarinen, O. H., Djurabekova, F. & Nordlund, K. A study on the elongation of embedded Au nanoclusters in SiO_2 by swift heavy ion irradiation using MD simulations. *Nucl. Instrum. Methods Phys. Res. B* **282**, 76 – 80 (2012). Ion Beam Synthesis and Modification of Nanostructured Materials and Surfaces.

2. Kluth, P. *et al.* Fine structure in swift heavy ion tracks in amorphous sio 2. *Phys. Rev. Lett.* **101**, 175503 (2008).

3. Pakarinen, O. H., Djurabekova, F., Nordlund, K., Kluth, P. & Ridgway, M. C. Molecular dynamics simulations of the structure of latent tracks in quartz and amorphous sio2. *Nucl. Instruments Methods Phys. Res. Sect. B: Beam Interactions with Mater. Atoms* **267**, 1456–1459 (2009).

4. Toulemonde, M. *et al.* Electronic sputtering of metals and insulators by swift heavy ions. *Nucl. Instruments Methods Phys. Res. Sect. B: Beam Interactions with Mater. Atoms* **212**, 346–357 (2003).

5. Ziegler, J. F., Ziegler, M. D. & Biersack, J. P. Srim–the stopping and range of ions in matter (2010). *Nucl. Instruments Methods Phys. Res. Sect. B: Beam Interactions with Mater. Atoms* **268**, 1818–1823 (2010).

6. Leino, A. A. *et al.* Swift heavy ion shape transformation of Au nanocrystals mediated by molten material flow and recrystallization. *Mater. Res. Lett.* **2**, 37–42 (2014).
